# Facilitators and Barriers to Social Engagement and Healthy Aging Among Older Adults

**DOI:** 10.1093/geroni/igaf122.3588

**Published:** 2025-12-31

**Authors:** Hin Wing Tse, Emily West, Catherine Sherk, Zoraida Garcia, Frida Ortega-Ruiz, Asma Maredia, Mattea Rodgers, Elizabeth Muñoz

**Affiliations:** The University of Texas at Austin, Austin, Texas, United States

## Abstract

Social relationships and community involvement are essential to successful aging, defined as maintaining mental and physical well-being in later life. The COVID-19 pandemic significantly increased social isolation among older adults in the United States and globally. This study utilized the World Café method, a Community-Based Participatory Research (CBPR) approach, to facilitate open and meaningful discussions in the community and foster collaborative learning. It explored the lived experiences of 41 older adults with diverse sociodemographic characteristics, including age (50 years and older), language (English or Spanish), and marital status (married, divorced, widowed, or never married), who were recruited from three Central Texas community centers. Discussions focused on patterns of engagement in social and physical activities, perceived links with mental health, and barriers and facilitators to participation in center activities before, during, and after COVID-19 restrictions. Data were audio-recorded, transcribed verbatim, and thematically analyzed using a deductive approach in Dedoose software. Emerging themes include: Physical activity improved mental health and reduced loneliness; social connection served as a strong motivator for community involvement and engagement in physical activity; lingering impacts of COVID-19, such as fear of illness, reduced program availability, and transportation challenges, continued to limit their participation. These results highlight the need for community-level capacity building to enhance engagement among older adults, particularly those most vulnerable to social disconnection.

